# Image analysis-based tumor infiltrating lymphocytes measurement predicts breast cancer pathologic complete response in SWOG S0800 neoadjuvant chemotherapy trial

**DOI:** 10.1038/s41523-023-00535-0

**Published:** 2023-05-13

**Authors:** Kristina A. Fanucci, Yalai Bai, Vasiliki Pelekanou, Zeina A. Nahleh, Saba Shafi, Sneha Burela, William E. Barlow, Priyanka Sharma, Alastair M. Thompson, Andrew K. Godwin, David L. Rimm, Gabriel N. Hortobagyi, Yihan Liu, Leona Wang, Wei Wei, Lajos Pusztai, Kim R. M. Blenman

**Affiliations:** 1 Department of Internal Medicine Section of Medical Oncology and Yale Cancer Center, Yale School of Medicine, 333 Cedar St, New Haven, CT 06520 USA; 2 Department of Pathology, Yale School of Medicine, 310 Cedar St, New Haven, CT 06520 USA; 3 Department of Hematology/Oncology, Cleveland Clinic Florida, Maroone Cancer Center, 2950 Cleveland Clinic Blvd, Weston, FL 33331 USA; 4 SWOG Statistics and Data Management Center, 1730 Minor Avenue Suite 1900, Seattle, WA 98101 USA; 5 Department of Medical Oncology, University of Kansas Medical Center, 3901 Rainbow Boulevard, Kansas City, KS 66160 USA; 6 Section of Breast Surgery, 1 Baylor Plaza, Baylor College of Medicine, Houston, TX 77030 USA; 7 Department of Breast Medical Oncology, MD Anderson Cancer Center, 1515 Holcombe Blvd, Houston, TX 77030 USA; 8 Department of Biostatistics, Yale School of Public Health, 60 College Street, New Haven, CT 06520 USA; 9 Department of Computer Science, Yale School of Engineering and Applied Science, 17 Hillhouse Avenue, New Haven, CT 06520 USA; 10 Present Address: Bayer Pharmaceuticals, 245 First St Cambridge Science Center 100 and 200 Floors 1 and 2, Cambridge, MA 02142 USA; 11 Present Address: Department of Pathology, Ohio State University, 6100 Optometry Clinic & Health Sciences Faculty Office Building, 1664 Neil Avenue, Columbus, OH 43210 USA

**Keywords:** Predictive markers, Breast cancer

## Abstract

We assessed the predictive value of an image analysis-based tumor-infiltrating lymphocytes (TILs) score for pathologic complete response (pCR) and event-free survival in breast cancer (BC). About 113 pretreatment samples were analyzed from patients with stage IIB-IIIC HER-2-negative BC randomized to neoadjuvant chemotherapy ± bevacizumab. TILs quantification was performed on full sections using QuPath open-source software with a convolutional neural network cell classifier (CNN11). We used easTILs% as a digital metric of TILs score defined as [sum of lymphocytes area (mm^2^)/stromal area(mm^2^)] × 100. Pathologist-read stromal TILs score (sTILs%) was determined following published guidelines. Mean pretreatment easTILs% was significantly higher in cases with pCR compared to residual disease (median 36.1 vs.14.8%, *p* < 0.001). We observed a strong positive correlation (*r* = 0.606, *p* < 0.0001) between easTILs% and sTILs%. The area under the prediction curve (AUC) was higher for easTILs% than sTILs%, 0.709 and 0.627, respectively. Image analysis-based TILs quantification is predictive of pCR in BC and had better response discrimination than pathologist-read sTILs%.

## Introduction

Image analysis-based tumor-infiltrating lymphocytes (TILs) quantification methods are being developed to eliminate the substantial reader-to-reader variation in TILs assessment that hinders clinical adoption of TILs as prognostic and chemotherapy response predictive markers in breast and other cancer types^1–4^. In melanoma, an image analysis-based assessment of TILs on hematoxylin and eosin (H&E) stained sections separated patients into prognostic cohorts more accurately than pathologist-read stromal TILs (sTILs) scores^1^. In triple-negative breast cancer (TNBC), high levels of TILs infiltration are also associated with better survival and increased pathologic complete response (pCR) to neoadjuvant (i.e., preoperative) chemotherapy^5–10^. While standardized rules for quantification of TILs in breast cancer have been developed^11^, the inter-observer variability in results continues to slow the adoption of TILs as routine prognostic and predictive markers^12^.

We previously showed that digital quantification of TILs using an open-source image analysis software, QuPath, and a convolutional neural network predictor algorithm (CNN11) could stratify patients with TNBC into distinct prognostic cohorts, and high digital TILs was independently associated with improved overall survival after adjustment of clinicopathological factors including stage, age, and histological grade of tumor^2^.

The S0800 trial was a randomized phase II neoadjuvant chemotherapy trial for patients with stage II and III HER-2-negative breast cancers, including both hormone receptor (HR) positive and negative tumors. Patients were randomly allocated (2:1:1) to three neoadjuvant chemotherapy arms:^1^ nab-paclitaxel with concurrent bevacizumab followed by AC;^2^ nab-paclitaxel followed by AC; or^3^ AC followed by nab-paclitaxel. The sequencing of taxane versus AC had no impact on the pCR rate, but the addition of bevacizumab improved the pCR rate from 21 to 36% (*p* = 0.019)^13^. Baseline core needle biopsies and posttreatment surgical resection specimens were collected prospectively for biomarker research. We previously reported that higher baseline immune gene expression^14^ and higher pathologist-read sTILs score^15^ were associated with higher pCR rates in this trial.

In the current study, we examined the chemotherapy response predictive and prognostic values of image analysis-based TILs assessment in pretreatment biopsies of the S0800 trial and compared its predictive performance to pathologist-read sTILs scores. We also assessed change in TILs in the subset of patients with residual cancer where paired pre- and post-treatment tissues were available.

## Results

**Fig. 1:**
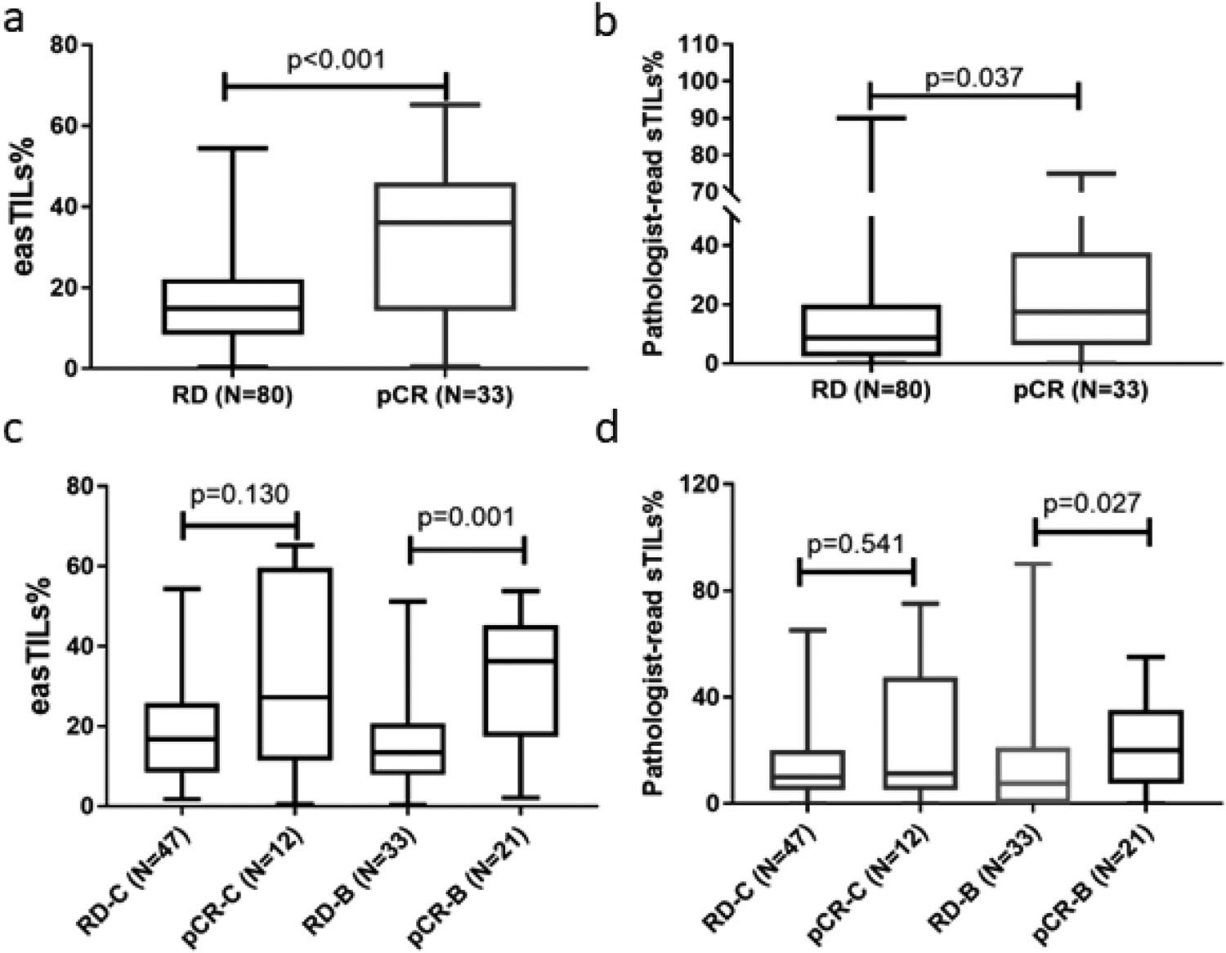
Pretreatment easTILs% and pathologist-read sTILs% by pCR and treatment status. **a** Pretreatment easTILs% by pCR status. **b** Pathologist-read sTILs% by pCR status. **c** Pretreatment easTILs% by pCR and treatment status. **d** Pretreatment sTILs% by pCR and treatment status. TILs tumor-infiltrating lymphocytes, easTILs% = [sum of lymphocytes area/stromal area] × 100, sTILs% pathologist assessment of stromal TILs, RD residual disease, pCR pathologic complete response, C control, chemotherapy alone, B bevacizumab + chemotherapy. Box and whiskers plots are shown with min to max whiskers that go down to the smallest value and up to the largest. Statistical analysis was performed using Mann–Whitney test.

**Fig. 2:**
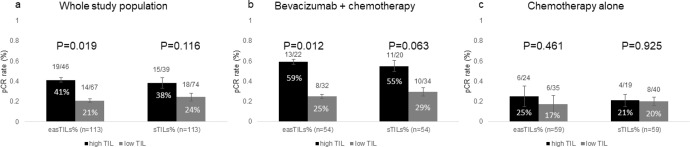
Pathologic complete response (pCR) rates by dichotomized high versus low tumor-infiltrating lymphocytes (TILs) status of pretreatment samples. **a** The whole study population, **b** Bevacizumab group, **c** chemotherapy alone group. easTILs% = [sum of lymphocytes area/stromal area] × 100, sTILs% pathologist assessment of stromal TILs, easTILs% high is defined as >19.9% and pathologist sTILs% high is defined as >20%. Error bars represent standard error. Statistical analysis was performed using the Chi-square test.

For the entire cohort, the mean, median, standard error, and interquartile range of easTILs% were 21.39, 17.02, 1.49, and 21.37%, respectively and of pathologist-read sTILs% were 17.85, 10.00, 1.87, and 17.50%, respectively. Patients with pCR had statistically higher pretreatment mean easTILs% compared to those with residual disease (RD) (median 36.1 vs. 14.8%, *p* < 0.001) (Fig. 1a). sTILs% was significantly higher in the pCR group compared to the RD group (median 17.5 vs. 8.8%, *p* = 0.037) (Fig. 1b). When the treatment arms were analyzed separately, significantly higher baseline easTILs% and sTILs% were seen in cancers with pCR in the bevacizumab arm, but not in the combined chemotherapy alone arms (Fig. 1c, d). However, the marker treatment interaction test did not demonstrate statistically significant differential predictive values for easTILs% or sTILs% by treatment type. When we dichotomized easTILs% into high (>19.9%) and low (≤19.9%) categories, the overall pCR rates were 41 and 21% (*p* = 0.019) in the easTILs% high and low groups, respectively (Fig. 2a). In the bevacizumab arm, the corresponding pCR rates were 59 and 25% (*p* = 0.012) (Fig. 2b) and in the chemotherapy alone arm the pCR rates were 25 and 17% (*p* = 0.46) in the easTILs% high and low groups, respectively (Fig. 2c). When we repeated this analysis using pathologist-read sTILs% with a cutoff of 20%, we obtained similar results as with easTILs% (Fig. 2a–c). However, in each comparison, the *p* values were lower for easTILs% than sTILs% on the same sample set, suggesting a greater ability to identify a difference. In the whole study population, multivariable logistic regression analysis including ER status, treatment arm, and disease type (IBC/LABC), easTILs% either as continuous or as categorical (high vs low) variable remained independently significantly predictive of pCR (continuous easTILs% *p* < 0.001; easTILs% high category *p* = 0.035) (Supplementary Tables 1, 2). There was no evidence that prognosis by easTILs% differed by hormone receptor status (Interaction: continuous *p* = 0.28; categorical *p* = 0.35).

**Fig. 3:**
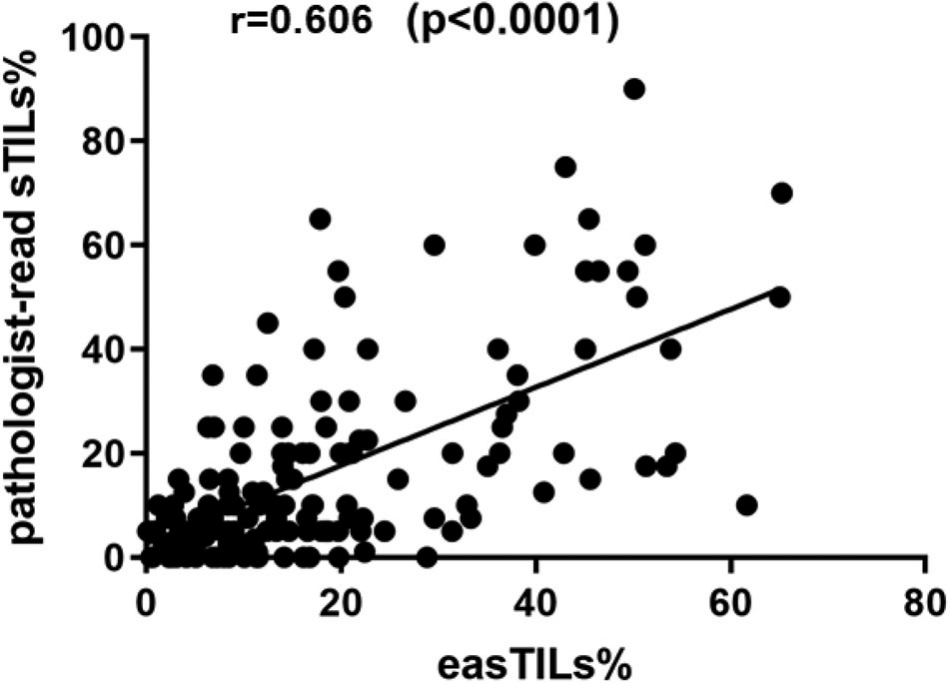
Correlation between pathologist-read sTILs% and image analysis-based easTILs% score. r Pearson correlation coefficient, TILs tumor-infiltrating lymphocytes. easTILs% [sum of lymphocytes area/stromal area] × 100. sTILs% pathologist assessment of stromal TILs. Statistical analysis was performed using the Pearson correlation coefficient.

**Fig. 4:**
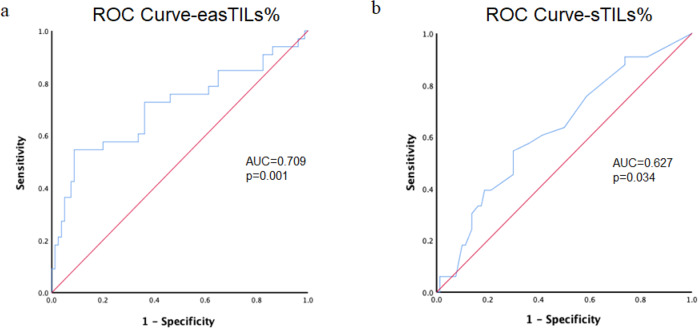
Receiver operator characteristic (ROC) curves for digital TILs and manual sTILs. **a** easTILs% and **b** sTILs%. AUC area under the ROC curve, easTILs% = [sum of lymphocytes area/stromal area] × 100, sTILs% pathologist assessment of stromal TILs, TILs tumor-infiltrating lymphocytes. Statistical analysis was performed using the DeLong test.

Pathologist-read sTILs% and digital easTILs% were positively and significantly correlated (*r* = 0.606, *p* < 0.0001) (Fig. 3). We compared the predictive performance of the two different scoring systems in receiver-operating characteristic (ROC) analysis in all patients included. The area under the ROC curves (AUCs) were 0.709 (95% CI 0.659–0.879) and 0.627 (95% CI 0.599–0.820) for easTILs% and sTILs%, respectively, although these AUCs are not statistically different (*p* = 0.11) (Fig. 4).

**Fig. 5:**
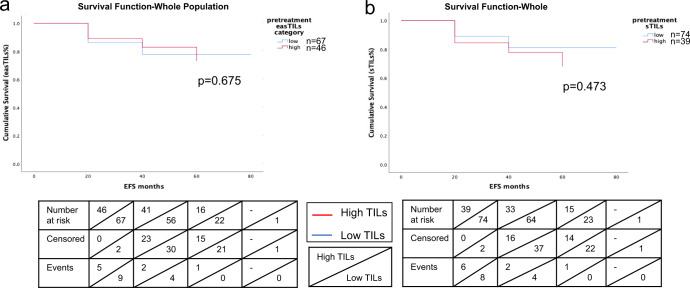
Kaplan–Meier curves of event-free survival (EFS) by high and low tumor-infiltrating lymphocytes (TILs) status. **a** EFS by easTILs% high (>19.9%) and low status in the whole study population. **b** EFS by sTILs% high (>20%) and low status in the whole study population. Statistical analysis was performed using the log-rank test.

**Fig. 6:**
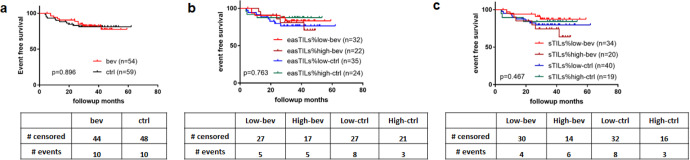
Kaplan–Meier curves of event-free survival (EFS). EFS by **a** treatment with or without bevacizumab, **b** easTILs% high or low and treatment status, **c** sTILs% high or low and treatment status. easTILs% dichotomized at the value of 19.9%. sTILs% dichotomized at the value of 20%. TILs tumor-infiltrating lymphocytes, easTILs% [sum of lymphocytes area/stromal area] × 100, sTILs% pathologist assessment of stromal TILs, RD residual disease, pCR pathologic complete response, ctrl control group with chemotherapy, bev bevacizumab + chemotherapy group. Statistical analysis was performed using the log-rank test.

Kaplan–Meier survival curves evaluating event-free survival (EFS) showed that patients with high easTILs% or high sTILs% had no significant difference in EFS compared to those with low TILs (Fig. 5). There was no difference in EFS comparing chemotherapy alone vs. chemotherapy plus bevacizumab groups (*p* = 0.90) (Fig. 6b), or when treatment group was further stratified by high and low easTILs% (*p* = 0.76) (Fig. 6c) or high and low sTILs% (*p* = 0.47) (Fig. 6d).

**Fig. 7:**
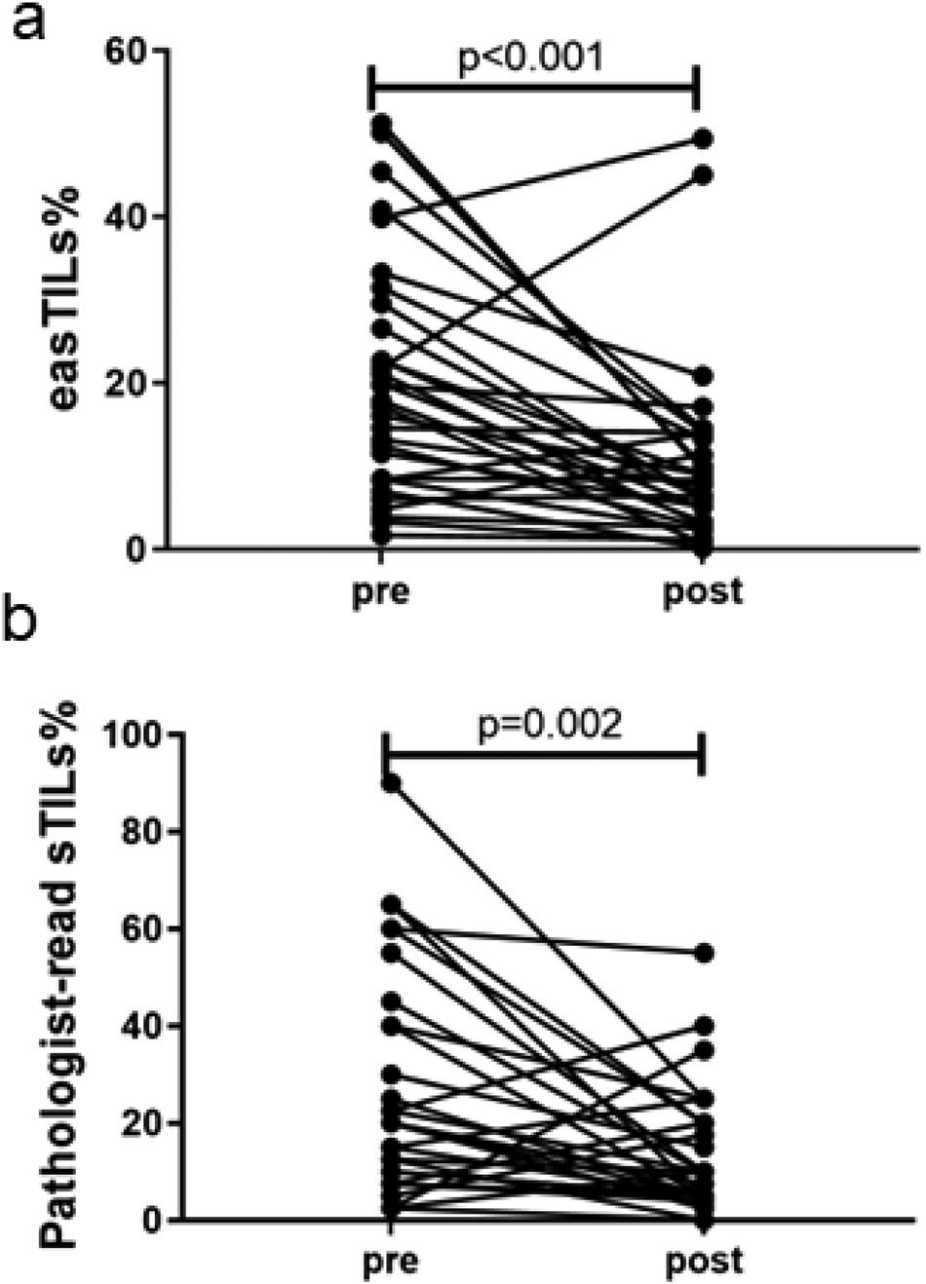
Changes in tumor-infiltrating lymphocytes (TILs) in paired pre- and post-treatment tissues of cases with residual disease. **a** Image analysis-based easTILs% scores, **b** Pathologist-read sTILs%. easTILs% [sum of lymphocytes area/stromal area] × 100. sTILs% pathologist assessment of stromal TILs. Statistical analysis was performed using the Wilcoxon matched-pairs signed rank test.

When TILs were compared between paired pre- and post-treatment tissues in patients with RD, we found that both easTILs% (pretreatment 20%, posttreatment 10%, *p* < 0.001) and sTILs% (pretreatment 26%, posttreatment 13%, *p* = 0.002) were significantly lower in residual cancer tissues compared to baseline (Fig. 7).

## Discussion

The association between immune cell infiltration of primary breast cancer and good prognosis has long been recognized, but despite attempts to standardize TILs scoring, inconsistencies in quantification limit the application of this biomarker in routine clinical care. Differences in preanalytical tissue processing contribute some variability to TILs assessment, but most of the variability arises from differences in pathologists’ scoring^16,17^. Image analysis-based TILs quantification holds promise for a more accurate and standardized assessment of TILs. In the current study, we assessed the predictive performance of a previously described breast cancer TILs quantification image analysis tool CNN11 implemented in QuPath, and compared its performance to pathologist-generated sTILs% results on pretreatment H&E-stained slides.

Our digital TILs metric, easTILs%, quantifies TILs density within the area of invasive cancer, counting both stromal and intratumoral lymphocytes correlated closely and significantly with pathologist-assessed sTILs%. Higher easTILs% was associated with a higher probability of pCR to neoadjuvant chemotherapy in the entire study population and in the bevacizumab plus chemotherapy arm of the trial. easTILs% high tumors also had a numerically higher pCR rate than easTILs% low cancers in the chemotherapy alone cohort, but this has not reached statistical significance. Marker treatment interaction test was not significant for differential treatment benefit by easTILs%. In multivariable analysis, easTILs% remained prognostic of pCR after adjustment for disease type, hormone receptor status, and randomization to bevacizumab. easTILs% had higher AUC than sTILs%, indicating a better discriminating ability.

We also examined pre- and post-treatment changes in easTILs% in paired samples in patients with residual disease. Cases with pCR were not included in this analysis because of the inability to consistently define the tumor bed for digital analysis, and we also previously demonstrated that in posttreatment tissues with pCR, the immune infiltration is largely resolved^15^. In the earlier analysis, based on pathologist-read sTILs%, we observed a trend to lower sTILs% in residual cancers compared to paired pretreatment tissues, but this difference has not reached statistical significance. In the current analysis, digital easTILs% was statistically significantly decreased in posttreatment tissues, consistent with a greater quantitative ability to detect differences with digital assessment.

Despite the promising performance of our digital TILs quantification method, there are several caveats. While QuPath is an open-source software, high-quality results require substantial human quality control because tissue pre-fixation time, fixation protocols, and microtome technique can change cell features and cause artifacts on tissue sections leading to poor performance of the classifier. False-positive TILs signal can be generated by apoptotic bodies, neutrophils, tissue artifact, and low-grade tumors with monotonously uniform nuclei^2,18^.

In summary, we demonstrated that a machine learning-derived digital measure of TILs correlates closely with pathologist-assessed sTILs score and is predictive of pCR in breast cancer. Digital TILs quantification had better outcome discrimination than pathologist-read stromal TILs score.

## Methods

### Patient cohorts and tissue preparation

**Fig. 8:**
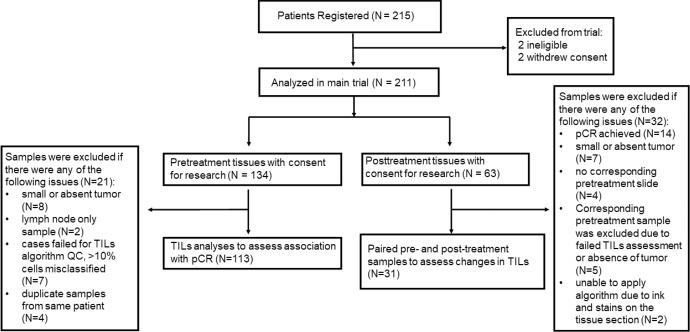
CONSORT diagram of samples used in the study. QC quality control, pCR pathologic complete response, RD residual invasive disease, TILs tumor-infiltrating lymphocyte.

**Fig. 9:**
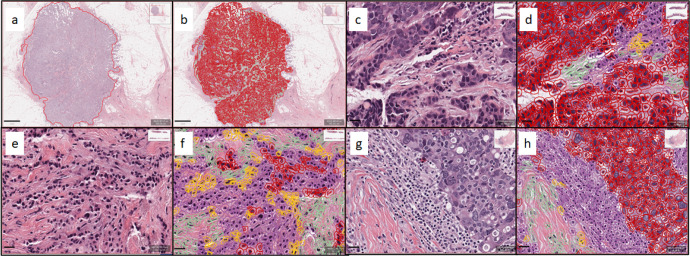
Representative hematoxylin and eosin (H&E) stained images and corresponding image analysis-based cell classification masks. **a** Invasive breast cancer region delineated at low magnification. **b** Cell classification mask applied within invasive breast cancer region. **c** Higher magnification image of invasive breast cancer within the delineated region. **d** Cell classification mask applied to the region of invasive breast cancer. **e** Representative image of invasive breast cancer within the delineated region. **f** Representative image of the case with inaccurate cell classification. **g** Representative image of invasive breast cancer within the delineated region. **h** Cell classification mask applied to the region of invasive breast cancer. Color code of cell classification mask: tumor cells (red), tumor-infiltrating lymphocytes (purple), fibroblasts (green), and others (yellow). Scale bar of (**a**, **b**): 1 mm; scale bar of (**c**–**h**): 20 μm.

Of the 215 patients registered in the S0800 trial, 134 patients had formalin fixed paraffin embedded pretreatment core needle biopsy tissues, 63 patients had posttreatment surgical resection tissues, including 59 paired pre- and post-treatment tissues, with written informed consent for future research (Fig. 8). Hematoxylin and eosin (H&E) stained full sections were used for TILs assessment. We were able to successfully generate digital TILs scores on 113 pre- and 31 post-treatment tissues, including 31 paired specimens. The remaining samples were excluded due to quality control failure, including lack of tumor on the section or artifact with ink or stains on tissue that interfered with image analysis; we also excluded slides where more than 10% of cells were misclassified according to the pathologist’s review (Fig. 9f). Patient characteristics of the S0800 trial population and the digital TILs quantification subpopulation were similar (Table 1). This study was approved by the Yale Cancer Center Human Investigations Committee. Two pathologists generated the pathologist-read sTILs% scores, which were defined as the percentage of invasive cancer stromal area occupied by mononuclear inflammatory cells as described in our previous report^15^. The reporting recommendations for tumor marker prognostic studies (REMARK) were followed^19^. This research is the result of data collected in clinical trial NCT00856492.

**Table 1:** Demographic and disease characteristics for the overall trial population and the digital TILs quantification subset.

	S0800	Digital TILs subgroup
Eligible and maintained consent	211	113
IBC or LABC		
IBC	24 (11.4%)	10 (8.8%)
LABC	187 (88.6%)	103 (91.2%)
HR status		
HR+ (ER+ and/or PR+)	144 (68.2%)	78 (69.0%)
HR− (ER− and PR−)	67 (31.8%)	35 (31.0%)
Randomized treatment		
No bevacizumab	113 (53.5%)	59 (52.2%)
bevacizumab	98 (46.5%)	54 (47.8%)
Primary outcome		
RD	152 (72.0%)	80 (70.8%)
pCR	59 (28.0%)	33 (29.2%)

### Digital image analysis

The Aperio ScanScope CS2 platform (Leica Biosystems, Wetzlar, Germany) was used to scan H&E-stained whole slides at 20x magnification and a pixel size of 0.4986 µm × 0.4986 µm. The QuPath version 0.1.2 open-source image analysis software (https://qupath.readthedocs.io/en/stable/) was used for digital data generation^1,2,20^. A convolutional neural network algorithm (CNN11) with eight hidden layers (maximum iterations: 100) that was previously trained to assign cells into one of four categories (i) tumor cells, (ii) lymphocytes, (iii) stromal cells, and (iv) other cells on stained sections was used to digitally quantify TILs^1,2,21^. The intensity of H&E staining varied from slide to slide and therefore, we recalibrated the H&E stain estimates for each digitized slide using the “estimate stain vectors” command in QuPath to produce normalized staining for each slide (Fig. 9). Watershed cell detection^22^ was used to segment the cells in the images with the following settings: Detection image: hematoxylin OD; requested pixel size: 0.5 µm; background radius: 8 µm; median filter radius: 0 µm; sigma: 1.5 µm; minimum cell area: 10 µm^2^; maximum cell area: 400 µm^2^; threshold: 0.1; maximum background intensity: 2. Cell expansion: 5 µm. To enhance classification accuracy, we also added smoothed object features at 25 and 50 µm radius to supplement the measurements of individual cells. The CNN11 tissue annotation consists of cell assignment to one of the four cell types described above, calculation of invasive tumor area (mm^2^), and calculation of area occupied by each cell type within the tumor area (mm^2^). The CNN11 algorithm has been deposited on GitHub. To digitally quantify TILs, we used the following formula: easTILs% = [sum of lymphocytes area (mm^2^)/stromal area (mm^2^)] × 100 where the stromal area (mm^2^) is the sum of all invasive tumor region areas (mm^2^) minus the sum of tumor cell area (mm^2^). easTILs%, therefore, represents the density of TILs per stromal area within invasive cancer and is the digital equivalent of pathologist scoring of stromal infiltrating lymphocytes as recommended by the International Immuno-Oncology Biomarker Working Group on Breast Cancer^11^. A subtle difference between sTILs% and easTILs% is that easTILs% includes intratumoral infiltrating lymphocytes, whereas sTILs% excludes these. Inflammatory infiltrates in the stroma of noninvasive lesions and normal breast structures were excluded from both the digital and pathologist-read TILs scores.

### Statistical analysis

All available specimens were used in this study, and the sample size was defined by tissue availability. The primary clinical outcome measure was pCR (ypT0/is ypN0). The Mann–Whitney test was used to investigate the association between pCR and easTILs% and sTILs%. Pearson correlation coefficient was used to assess the correlation between pathologist-read sTILs% with paired easTILs%. We also dichotomized easTILs% into low and high categories using our previously published optimal cut point of 19.9%^2^ and compared pCR rates in the two groups using the Chi-square test. The secondary clinical endpoint was EFS, defined as the time from registration to progression prior to surgery, recurrence post-surgery, or death from any cause. Patients without an event were censored at the time of the last known follow-up. The Kaplan–Meier method and log-rank test were used to plot and compare survival curves implemented in the GraphPad Prism software (GraphPad Software Inc., San Diego, CA) and IBM SPSS Statistics for Macintosh version 26 (IBM Corp., Armonk, N.Y., USA). The two control arms were combined to compare to the bevacizumab arm. The predictive performance of easTILs% and sTILs% were compared using ROC analysis and AUC values implemented in R (version 4.1.0). The difference in AUC was tested using the DeLong test. Change in easTILs% in paired pre- and post-treatment samples was compared by Wilcoxon matched-pairs signed rank test. In all statistical analysis, the level of significance was set at *p* < 0.05. Multivariable logistic regression models were used to examine predictive factors (ER status, treatment arm, disease type, and easTILs%) for pCR jointly.

### Reporting summary

Further information on research design is available in the Nature Research Reporting Summary linked to this article.

## Supplementary information

Supplemental Material

Reporting Summary

## Data Availability

The data from which the results of this study are calculated are available upon request. The CNN11 algorithm is deposited on GitHub: https://github.com/Yalaibai/Automated_QuPath_TIL_-Classifier_for-TNBC.git. Digitalized images used in this study were deposited into the National Institutes of Health National Cancer Institute The Cancer Imaging Archive (TCIA) at 10.7937/awa3-sc85. The clinical data is deposited on National Cancer Institute NCTN/NCORP Data Archive (https://nctn-data-archive.nci.nih.gov) under NCT00856492-D1.
