# Supplementary material for: Image analysis-based tumor infiltrating lymphocytes measurement predicts breast cancer pathologic complete response in SWOG S0800 neoadjuvant chemotherapy trial
Source: NPJ Breast Cancer. 2023 May 13;9:38. doi: 10.1038/s41523-023-00535-0 (PMC10182981; doi:10.1038/s41523-023-00535-0)
Supplement: Supplementary file 1 — Supplemental Material [file 41523_2023_535_MOESM1_ESM.pdf]

Supplementary Table 1: Multivariable model of pCR including high/low easTILs%.

	Odds Ratio	Standard Error	Z	P > |z|	(95% CI)
High/low easTILs%	2.60	1.18	2.11	0.035	1.07 – 6.33
Bev vs. control	2.61	1.18	2.13	0.033	1.08 – 6.31
HR positive	0.53	0.25	-1.33	0.185	0.21 – 1.35
LABC vs. IBC	4.73	5.16	1.43	0.154	0.56 – 40.11

pCR = pathologic complete response; CI = confidence interval; Bev = bevacizumab; HR = hormone receptor; LABC = locally advanced breast cancer; IBC = inflammatory breast cancer. Statistical analysis performed using multivariable logistic regression.

Supplementary Table 2: Multivariable model of pCR including easTILs% score.

	Odds Ratio	Standard Error	Z	P > |z|	(95% CI)
easTILs% score	1.06	0.016	3.73	0.000	1.03 – 1.09
Bev vs. control	2.93	1.42	2.21	0.027	1.13 – 7.58
HR positive	0.67	0.34	-0.80	0.425	0.25 – 1.80
LABC vs. IBC	4.20	4.71	1.28	0.201	0.47 – 37.89

pCR = pathologic complete response; CI = confidence interval; Bev = bevacizumab; HR = hormone receptor; LABC = locally advanced breast cancer; IBC = inflammatory breast cancer. Statistical analysis performed using multivariable logistic regression.
