# Supplementary material for: Image analysis-based tumor infiltrating lymphocytes measurement predicts breast cancer pathologic complete response in SWOG S0800 neoadjuvant chemotherapy trial
Source: NPJ Breast Cancer. 2023 May 13;9:38. doi: 10.1038/s41523-023-00535-0 (PMC10182981; doi:10.1038/s41523-023-00535-0)
Supplement: Supplementary file 2 — Reporting Summary [file 41523_2023_535_MOESM2_ESM.pdf]

Corresponding author(s): Dr Kim RM Blenman; DBPR NPJBCANCER-01941

Last updated by author(s): Apr 1, 2023

Reporting Summary

Nature Portfolio wishes to improve the reproducibility of the work that we publish. This form provides structure for consistency and transparency in reporting. For further information on Nature Portfolio policies, see our Editorial Policies and the Editorial Policy Checklist.

Statistics

For all statistical analyses, confirm that the following items are present in the figure legend, table legend, main text, or Methods section.

n/a	Confirmed	
☐	☒	The exact sample size (*n*) for each experimental group/condition, given as a discrete number and unit of measurement
☐	☒	A statement on whether measurements were taken from distinct samples or whether the same sample was measured repeatedly
☐	☒	The statistical test(s) used AND whether they are one- or two-sided<br>*Only common tests should be described solely by name; describe more complex techniques in the Methods section.*
☐	☒	A description of all covariates tested
☐	☒	A description of any assumptions or corrections, such as tests of normality and adjustment for multiple comparisons
☐	☒	A full description of the statistical parameters including central tendency (e.g. means) or other basic estimates (e.g. regression coefficient) AND variation (e.g. standard deviation) or associated estimates of uncertainty (e.g. confidence intervals)
☐	☒	For null hypothesis testing, the test statistic (e.g. F, t, r) with confidence intervals, effect sizes, degrees of freedom and P value noted<br>*Give P values as exact values whenever suitable.*
☐	☒	For Bayesian analysis, information on the choice of priors and Markov chain Monte Carlo settings
☐	☒	For hierarchical and complex designs, identification of the appropriate level for tests and full reporting of outcomes
☐	☒	Estimates of effect sizes (e.g. Cohen's *d*, Pearson's *r*), indicating how they were calculated

Our web collection on statistics for biologists contains articles on many of the points above.

Software and code

Policy information about availability of computer code

Data collection	The QuPath version 0.1.2 open-source image analysis software (https://qupath.readthedocs.io/en/stable/) was used for digital data generation (1, 2, 20) with convolutional neural network algorithm (CNN11).
Data analysis	GraphPad Prism software (GraphPad software Inc., San Diego, CA) and IBM SPSS Statistics for Macintosh version 26 (IBM Corp., Armonk, N.Y., USA). R (version 4.1.0) was also used.

For manuscripts utilizing custom algorithms or software that are central to the research but not yet described in published literature, software must be made available to editors and reviewers. We strongly encourage code deposition in a community repository (e.g. GitHub). See the Nature Portfolio guidelines for submitting code & software for further information.

Data

Policy information about availability of data

All manuscripts must include a data availability statement. This statement should provide the following information, where applicable:

- Accession codes, unique identifiers, or web links for publicly available datasets
- A description of any restrictions on data availability
- For clinical datasets or third party data, please ensure that the statement adheres to our policy

The data from which the results of this study are calculated are available upon request. The CNN11 algorithm is deposited on GitHub: https://github.com/Yalaibai/Automated_QuPath_TIL_-Classifier_for-TNBC.git. Digitalized images used in this study were deposited into the National Institutes of Health National Cancer

Institute The Cancer Imaging Archive (TCIA) at DOI: https://doi.org/10.7937/awa3-sc85. The clinical data is deposited on National Cancer Institute NCTN/NCORP Data Archive (https://nctn-data-archive.nci.nih.gov) under NCT00856492-D1.

Human research participants

Policy information about studies involving human research participants and Sex and Gender in Research.

Reporting on sex and gender	Data individuals of female sex were used in this study. Sex data was reported in the original study (Breast Cancer Res Treat (2016) 158:485–495; DOI 10.1007/s10549-016-3889-6).
Population characteristics	Breast cancer type (IBC; LABC), HR status, randomization treatment, and primary outcome were the covariates used in this study.
Recruitment	No patient recruitment was required for this study. Existing data was used.
Ethics oversight	This study was approved by the Yale Cancer Center Human Investigations Committee.

Note that full information on the approval of the study protocol must also be provided in the manuscript.

Field-specific reporting

Please select the one below that is the best fit for your research. If you are not sure, read the appropriate sections before making your selection.

☒ Life sciences ☐ Behavioural & social sciences ☐ Ecological, evolutionary & environmental sciences

For a reference copy of the document with all sections, see nature.com/documents/nr-reporting-summary-flat.pdf

Life sciences study design

All studies must disclose on these points even when the disclosure is negative.

Sample size	No sample size calculation was performed. The sample size was determined based on the availability of the data from the original clinical trial.
Data exclusions	Of the original 215 patients in the clinical trial, we generated digital TILs scores on 113 pre- and 31 post-treatment tissues, including 31 paired specimens. The remaining samples were excluded due to quality control failure including lack of tumor on the section or artifact with ink or stains on tissue that interfered with image analysis. We also excluded slides where more than 10% of cells were misclassified according to the pathologist's review.
Replication	This study is a validation of our CNN11 convolutional neural network algorithm that has been used in several studies including breast cancer.<br>1. Acs B, Ahmed FS, Gupta S, Wong PF, Gartrell RD, Sarin Pradhan J, et al. An open source automated tumor infiltrating lymphocyte algorithm for prognosis in melanoma. Nat Commun. 2019;10(1):5440.<br>2. Bai Y, Cole K, Martinez-Morilla S, Ahmed FS, Zugazagoitia J, Staaf J, et al. An Open-Source, Automated Tumor-Infiltrating Lymphocyte Algorithm for Prognosis in Triple-Negative Breast Cancer. Clin Cancer Res. 2021;27(20):5557-65.
Randomization	Multivariable logistic regression models were used to examine predictive factors (ER status, treatment arm, disease type, and easTILs%) for pCR jointly.
Blinding	Investigators were blinded for the data collection but were not blinded for the analysis of the data.

Reporting for specific materials, systems and methods

We require information from authors about some types of materials, experimental systems and methods used in many studies. Here, indicate whether each material, system or method listed is relevant to your study. If you are not sure if a list item applies to your research, read the appropriate section before selecting a response.

Materials & experimental systems

n/a	Involved in the study
☒	☐ Antibodies
☒	☐ Eukaryotic cell lines
☒	☐ Palaeontology and archaeology
☒	☐ Animals and other organisms
☐	☒ Clinical data
☒	☐ Dual use research of concern

Methods

n/a	Involved in the study
☒	☐ ChIP-seq
☒	☐ Flow cytometry
☒	☐ MRI-based neuroimaging

Clinical data

Policy information about clinical studies

All manuscripts should comply with the ICMJE guidelines for publication of clinical research and a completed CONSORT checklist must be included with all submissions.

Clinical trial registration	NCT00856492
Study protocol	The full study protocol can be obtained from the NCI Southwest Oncology Group.
Data collection	Data was collected from NCI Southwest Oncology Group existing study SWOG S0800.
Outcomes	Outcomes were generated as part of the original clinical trial (Breast Cancer Res Treat (2016) 158:485–495; DOI 10.1007/s10549-016-3889-6).
